# Setting research priorities to achieve long-term health targets in Iran

**DOI:** 10.7189/jogh.08.020702

**Published:** 2018-12

**Authors:** Parisa Mansoori, Reza Majdzadeh, Zhaleh Abdi, Igor Rudan, Kit Yee Chan, The Iranian CHNRI Health Research Priority Setting Group, Mohsen Aarabi, Elham Ahmadnezhad, Shirin Ahmadnia, Shahin Akhondzadeh, Ali Azin, Fereidoun Azizi, Reza Dehnavieh, Hassan Eini-Zinab, Farshad Farzadfar, Mohammad Hosein Farzaei, Mostafa Ghanei, AliAkbar Haghdoost, Sedigheh Hantoushzadeh, Gholamreza Heydari, Hassan Joulaei, Naser Kalantari, Roya Kelishadi, Ardeshir Khosravi, Bagher Larijani, Amir Hossein Mahvi, Ali Reza Massah Bavani, Alireza Mesdaghinia, Azarakhsh Mokri, Ali Montazeri, Ehsan Mostafavi, Seyed Abbas Motevalian, Kazem Naddafi, Shekoufeh Nikfar, Seyed Ali Nojoumi, Maryam Noroozian, Alireza Olyaeemanesh, Nasrin Omidvar, Abbas Ostadtaghizadeh, Farshad Pourmalek, Roja Rahimi, Afarin Rahimi-Movaghar, Arash Rashidian, Emran Razaghi, Homayoun Sadeghi-Bazargani, Gholamhosain Salehi Zalani, Hamid Soori, Jafar Sadegh Tabrizi, AbouAli Vedadhir, Bahareh Yazdizadeh, Masud Yunesian, Mehdi Zare

**Affiliations:** 1 Centre for Global Health Research, Usher Institute of Population Health Sciences and Informatics, University of Edinburgh, Edinburgh, UK; 2 Knowledge Utilization Research Center, Tehran University of Medical Sciences, Tehran, Iran; 3 Community Based Participatory Research Center, Tehran University of Medical Sciences, Tehran, Iran; 4 School of Public Health, Tehran University of Medical Sciences, Tehran, Iran; 5 National Institute of Health Research (NIHR), Tehran University of Medical Sciences, Tehran, Iran; 6 Nossal Institute for Global Health, University of Melbourne, Australia; 7 Department of Family Medicine, School of Medicine, Mazandaran University of Medical Sciences, Sari, Iran; 8 Diabetic Research Center, Cancer Institute, Mazandaran University of Medical Sciences, Sari, Iran; 9 Allameh Tabataba'i University, Tehran, Iran; 10 Psychiatric Research Center, Roozbeh Hospital, Tehran University of Medical Sciences, Tehran, Iran; 11 Reproductive Biotechnology Research Center, Avicenna Research Institute (ACECR), Tehran, Iran; 12 Endocrine Research Center, Research Institute for Endocrine Sciences, Shahid Beheshti University of Medical Sciences, Tehran, Iran; 13 Health Services Management Research Center, Institute for Future Studies in Health, Kerman University of Medical Sciences, Kerman, Iran; 14 Shahid Beheshti University of Medical Sciences, Tehran, Iran; 15 Non-Communicable Diseases Research Center, Endocrinology and Metabolism Population Sciences Institute, Tehran University of Medical Sciences, Tehran, Iran; 16 Pharmaceutical Sciences Research Center, Kermanshah University of Medical Sciences, Kermanshah, Iran; 17 Chemical Injuries Research Center, Systems Biology and Poisoning Institute, Tehran, Iran; 18 HIV/STI Surveillance Research Center, and WHO Collaborating Center for HIV Surveillance, Institute for Futures Studies in Health, Kerman University of Medical Sciences, Kerman, Iran; 19 Maternal, Fetal and Neonatal Research Center, Tehran University of Medical Sciences (TUMS), Tehran, Iran; 20 Tobacco Prevention & Control Research Center, National Research Institute of TB & Lung Diseases, Shahid Beheshti University of Medical Sciences Tehran, Iran; 21 Shiraz HIV/AIDS Research Center, Institute of Health, Shiraz University of Medical Sciences, Shiraz, Iran; 22 National Institute and Faculty of Nutrition Sciences and Food Technology, Department of Community Nutrition, Shahid Beheshti University of Medical Sciences; 23 Child Growth and Development Research Center, Research Institute for Primordial Prevention of Non-Communicable Disease, Isfahan University of Medical Sciences, Isfahan, Iran; 24 Deputy for Public Health, Ministry of Health and Medical Education, Tehran, Iran; 25 Endocrinology and Metabolism Research Center, Endocrinology and Metabolism Clinical Sciences Institute, Tehran University of Medical Sciences, Tehran, Iran; 26 Center for Solid Waste Research, Institute for Environmental Research, Tehran University of Medical Sciences, Tehran, Iran; 27 Department of Irrigation and Drainage Engineering, Aburaihan Campus, University of Tehran, Pakdasht, Iran; 28 School of Public Health, Tehran University of Medical Sciences, Institute for Environmental Research, Tehran University of Medical Sciences, Tehran, Iran; 29 Iranian National Center for Addiction Studies (INCAS), Tehran University of Medical Sciences, Tehran, Iran; 30 Population Health Research Group, Health Metrics Research Center, Institute for Health Sciences Research, ACECR. Tehran, Iran; 31 Department of Epidemiology and Biostatistics, Research Centre for Emerging and Reemerging Infectious Diseases, Pasteur Institute of Iran, Tehran, Iran; 32 Department of Epidemiology, School of Public Health, Iran University of Medical Sciences, Tehran, Iran; 33 Center for Air Pollution Research (CAPR), Institute for Environmental Research (IER), Tehran University of Medical Sciences, Tehran, Iran; 34 Department of Environmental Health Engineering, School of Public Health, Tehran University of Medical Sciences, Tehran, Iran; 35 Department of Pharmacoeconomics and Pharmaceutical Administration, Faculty of Pharmacy and Pharmaceutical Management and Economics Research Center, Tehran University of Medical Sciences, Tehran, Iran; 36 Microbiology Research Center, Pasteur Institute of Iran, Tehran, Iran; 37 Memory and Behavioral Neurology Division, Department of Psychiatry, Tehran University of Medical Sciences (TUMS), Iran; 38 Health Equity Research Centre, Tehran University of Medical Sciences, Tehran Iran; 39 Department of Community Nutrition, National Nutrition and Food Technology Research Institute, Faculty of Nutrition, Tehran, Iran; 40 Department of Disaster Public Health, School of Public Health, Tehran University of Medical sciences, Tehran, Iran; 41 School of Population and Public Health, University of British Columbia, Vancouver, Canada; 42 Department of Pharmacy in Persian Medicine, School of Persian Medicine, Tehran University of Medical Sciences, Tehran, Iran; 43 Director of Information, Evidence and Research, Eastern Mediterranean Regional Office, World Health Organization, Cairo, Egypt; 44 Department of Psychiatry, School of Medicine, Tehran University of Medical Sciences, Tehran, Iran; 45 Road Traffic Injury Research Center, Tabriz University of Medical Sciences, Tabriz, Iran; 46 Ministry of Health and Medical Education, Tehran, Iran; 47 Safety Promotion and Injury Prevention Research Center, Shahid Beheshti University of Medical Sciences; 48 Tabriz Health Services Management Research Center, Health, Management and Safety Promotion Research Institute, Tabriz University of Medical Sciences, Tabriz, Iran; 49 Department of Anthropology, Faculty of Social Sciences, University of Tehran, Tehran, Iran; 50 UCL Department of Science and Technology Studies, University College London, Gower Street, London, UK; 51 Department of Research Methodology and Data Analysis, Institute for Environmental Research, Tehran University of Medical Sciences, Tehran, Iran; 52 Engineering Seismology Department, International Institute of Earthquake Engineering and Seismology (IIEES), Tehran, Iran; * Joint corresponding authors

## Abstract

**Background:**

In 2015, it was estimated that the burden of disease in Iran comprised of 19 million disability-adjusted life years (DALYs), 74% of which were due to non-communicable diseases (NCDs). The observed leading causes of death were cardiovascular diseases (41.9%), neoplasms (14.9%), and road traffic injuries (7.4%). Even so, the health research investment in Iran continues to remain limited. This study aims to identify national health research priorities in Iran for the next five years to assist the efficient use of resources towards achieving the long-term health targets.

**Methods:**

Adapting the Child Health and Nutrition Research Initiative (CHNRI) method, this study engaged 48 prominent Iranian academic leaders in the areas related to Iran’s long-term health targets, a group of research funders and policy makers, and 68 stakeholders from the wider society. 128 proposed research questions were scored independently using a set of five criteria: feasibility, impact on health, impact on economy, capacity building, and equity.

**Findings:**

The top-10 priorities were focused on the research questions relating to: health insurance system reforms to improve equity; integration of NCDs prevention strategy into primary health care; cost-effective population-level interventions for NCDs and road traffic injury prevention; tailoring medical qualifications; epidemiological assessment of NCDs by geographic areas; equality in the distribution of health resources and services; current and future common health problems in Iran’s elderly and strategies to reduce their economic burden; the status of antibiotic resistance in Iran and strategies to promote rational use of antibiotics; the health impacts of water crisis; and research to replace the physician-centered health system with a team-based one.

**Conclusions:**

These findings highlight consensus amongst various prominent Iranian researchers and stakeholders over the research priorities that require investment to generate information and knowledge relevant to the national health targets and policies. The exercise should assist in addressing the knowledge gaps to support both the National General Health Policies by 2025 and the health targets of the United Nations’ Sustainable Development Goals by 2030.

Iran has achieved substantial health gains in recent decades, such as reduction of under-5 mortality from 67.4 deaths per 1000 live births in 1990 to 18.8 in 2015, and halving maternal mortality within the same period [1]. Even so, it was estimated that the total number of disability-adjusted life years (DALYs) in Iran in 2015 exceeded 19 million DALYs [2]. Although the country continues to struggle with infectious diseases [3], the greatest contemporary challenge is the transition to non-communicable diseases (NCDs) [2], contributing to 74% of DALYs in Iran [2]. The observed leading causes of death were cardiovascular diseases (41.9%), neoplasms (14.9%), and road traffic injuries (7.4%) [4].

While Iran’s investment in health care had risen up to 8.9% of its Gross Domestic Product (GDP) by 2015, investment in research remains limited [5]. Investment in Research and Development has rarely surpassed 0.6% of Iran’s GDP [5], and only a fraction of this amount relates to health. Regardless of this limited investment, the past three decades have seen a substantial growth in the number of health research publications from Iran that are being abstracted in international bibliographic databases [6].

Despite the impressive surge of research publications, various studies have suggested that Iran’s research activities in health are not well aligned with the structure of the country’s disease burden and the health needs of its population [7-9]. These studies propose that a key problem might lie in the absence of a systematic process of health research prioritization that engages both the knowledge producers (ie, researchers) and users (ie, policy makers and members of the wider society) [8].

There seems to be a substantial need to a proper prioritization process to clarify the research priorities and align them with Iran’s national and international health agendas. The Child Health and Nutrition Research Initiative (CHNRI) method is a systematic and transparent health research priority-setting process that engages researchers, research funders and policy makers, and stakeholders [10,11]. This method has achieved a broad recognition and application worldwide over the last decade [11,12]. This study aims to adapt the CHNRI method to identify health research priorities in Iran to assist the efficient use of resources towards achieving the long-term health targets. It should also provide a model to other low- and middle-income countries on how to effectively adapt this prioritization process to improve funding allocation for health research.

## METHODS

This study (conducted between July-December 2017) used the CHNRI method (see **Box 1**). The invited experts generated and systematically ranked research questions (RQs) that could potentially assist Iran in achieving its long-term health targets, as outlined in the Iran’s General Health Policies (GHPs) and the United Nations’ Sustainable Development Goals (SDGs). The priorities were identified for the next five years to be aligned with Iran’s major national plans. Detailed information about different components of the context of this exercise and the process in which the context was defined is provided in Table S1 in **Online Supplementary Document(Online Supplementary Document)**, and a full list of the included health targets is available in Table S2 in **Online Supplementary Document(Online Supplementary Document)**.

Box 1 **The CHNRI method for setting research priorities**

The CHNRI method uses the principle of “wisdom of the crowds” to score ideas against a pre-defined set of criteria [11]. This enables funders and policy makers to view the strengths, the weaknesses, and relative ranking of each proposed research idea based on submitted opinions of a larger number of experts [13]. This method allows researchers to independently generate and score research questions, thus limits the influence of individuals on the rest of the group. It also involves funders, policy makers, and other stakeholders at an early stage of the process, ensuring their ownership in the outcomes [13]. Other recognized advantages of the CHNRI method include its systematic nature, transparency and replicability, clearly defined context and criteria, a structured way of obtaining information, informative and intuitive quantitative outputs, and studying the level of agreement over each proposed research idea [10,11,13]. The CHNRI method has thus far been implemented in more than 50 studies led by multilateral organizations, eg, WHO and UNICEF, national governments, eg, South Africa and India, and funders such as The Gates Foundation, to set research priorities in areas ranging from the reduction of global child mortality, dementia, or disability to the efficient execution of national health plans (eg, in China) [11,12].

Sixty-eight experts in areas relevant to Iran’s long-term health targets were identified through a combination of the following approaches: (i) a systematic search of international bibliometric databases (Scopus and Web of Science) for prolific researchers with Iranian affiliations in the fields relevant to the included health targets; (ii) a systematic search of the web in Persian for experts who have held leading academic roles in Iranian scientific societies and or universities, or have held scientific roles in executive organizations, eg, the Ministry of Health and Medical Education; (iii) “snowballing”, by asking the identified experts to nominate further persons. The final list of experts were checked and overseen by RM (Reza Majdzadeh) and ZA (Zhaleh Abdi) to ensure that the list had a comprehensive coverage of experts.

In the first instance, the identified experts were invited to participate in the exercise by independently generating three to five RQs. Fifty experts (73.5% of all 68 invitees) agreed to participate and generated a total of 251 RQs. The steering committee of this exercise (ie, the first 5 authors of this paper) retained 128 RQs after removing apparent duplicate questions and merging very similar ones. The consolidated list of 128 RQs, the five criteria, and scoring instructions were sent to the original 68 experts with an invitation to score the RQs. The 5 scoring criteria (presented in **Table 1**) were chosen and defined specifically for this exercise using input from a management group of nine persons which included Iranian health research policy makers and funders, and the steering committee. The process in which the criteria were selected is described in Table S3 in **Online Supplementary Document(Online Supplementary Document)**.

The CHNRI method provides four response options for scoring: 0; 0.5; 1; or leaving blank in case the expert does not feel sufficiently informed to respond. However, within the steering committee of this exercise, it was agreed that typing a 3-digit response—ie, “0.5”— may not seem convenient, hence the instructions were adapted as follows: scoring “3” for “yes”; “2” for “informed but undecided”; “1” for “no”; and “0” for “insufficiently informed”. For calculating the results, all responses were re-coded to the standard CHNRI scoring system. In total, 48 of the initial 68 invited experts (70.6%) completed scoring. An overview of experts’ identification process, background information, and participation is provided in Tables S4 and S5 in **Online Supplementary Document(Online Supplementary Document)**.

To ensure that the prioritization exercise considered views of a wider group of stakeholders, relative weights for each of the five criteria were calculated using the input from a larger reference group (see **Box 2**).

**Table 1:** List of the included five criteria and the agreed definition for each

Number	Criterion	Definition
**1**	Feasibility	(1) There is sufficient capacity (eg, data infrastructure, laboratory equipment) to carry out this research; (2) It is possible to provide training for the staff who would undertake this research; (3) This research can be conducted in an ethical way and produce informative results within the next five years
**2**	Impact on health	Results of this research have high potential to improve health by: (1) reducing disease incidence and/or prevalence; (2) reducing social, environmental, and/or individual risk factors of ill-health; (3) shaping future health planning and implementation; (4) improving health services delivery by improving acceptability, accessibility, suitability, efficiency, efficacy, effectiveness, and/or safety of treatment or service; and/or (5) improving societal and system preparedness for future health challenges
**3**	Impact on economy	Results of this research have high potential in: (1) having direct effect on the production of materials or consumer services; (2) optimising the earlier goods and/or products (increasing quality and/or reducing production costs); (3) creating knowledge-based entrepreneurship; (4) decreasing days of work missed due to illness or disability for patients and caregivers; (5) reducing opportunity costs for patients and caregivers; (6) reducing impact on direct patient costs as well as health and welfare systems; and/or (7) reducing caregiver burden and its associated financial costs (including health care costs for caregivers)
**4**	Capacity building	Results of this research have high potential to lead to: (1) education and training in Iran's human resources; (2) the acquisition of new skills by the research team; and/or (3) investment to improve research facilities/amenities where the study will be undertaken, eg, purchasing software/equipment
**5**	Equity	Results of this research have high potential to: (1) lead to interventions or services that will be accessible and affordable to everyone, including members of vulnerable groups; (2) lead to policy, plans, interventions or services that could reduce health inequality. This could be achieved by policies or interventions that target and empower vulnerable groups to reduce risk and disease exposures and/or improve access to services or interventions.

To rank the RQs, “weighted research priority scores” were calculated. This score took into account the average score provided by the experts to each RQ across the 5 criteria, and the weights assigned by the stakeholders to each criterion. The average scores – that initially were between 0 and 1 – were multiplied by 100 to provide scores in a range between 0 and 100. The RQs were also ranked based on the scores for each criterion. Average expert agreement (AEA) – ie, the level of agreement among the scorers – on each RQ was also computed as the frequency of the mode (ie, the most common score divided by the total number of scores). All the independent responses of scorers, calculations that led to the final scores, weights, and AEA are provided in **Online Supplementary Document(Online Supplementary Document),** in a Microsoft Excel (Microsoft Inc, Seattle WA, USA) file.

All of the original materials and correspondences with participants in this exercise were in Persian, and the ones that required further discussion within the steering committee were translated into English by one of the authors (Parisa Mansoori). To ensure that the researchers felt free to express their opinions while generating and/or scoring RQs, they were informed that nobody outside the steering committee of the exercise would be able to link their input to their real names.

## RESULTS

In total, 128 RQs were systematically scored by 48 experts against five criteria. Sixty-eight representatives from stakeholder groups provided weights to the five criteria (see **Box 2**). The weighted Research Priority Scores (wRPS) ranged from 84.5% to 28.5%. **Table 2** shows the top 10 priorities, the wRPSs, the score that each priority has received for each criterion, and the AEA. Full list of the ranked 128 RQs is provided in **Online Supplementary Document(Online Supplementary Document),** Excel file. Ninety-two of the 128 RQs (71.8%) fell entirely or partially under Health Policy and Systems Research (HPSR – using the definition of the Alliance for Health Policy and Systems Research [15]). Forty-five RQs addressed specific causes of morbidity, mortality, and/or interventions for their diagnosis, prevention, and/or management. Sixteen RQs fell into the category of “Planetary Health” (ie, addressing human health effects of accelerating environmental changes – the term was coined in 2014 [16]). Other themes that were found across the 128 RQs were traditional Iranian medicine and Iranian-Islamic values (9 RQs); medical education, science, and innovation (6 RQs); and community participation (5 RQs).

Box 2 **Engaging stakeholders**

Engaging laypersons, frontline health workers, and patients in health research prioritization processes is highly recommended because these people have their own specialized knowledge and a real stake in the outcome of the process [14]. Participants in the stakeholders’ group in this exercise were identified using snowballing in addition to sharing a public invitation in online forums of patients and health care professionals in Iran. The online platform that was used for this purpose was an instant messaging service called Telegram app. Telegram app was the most popular instant messaging service among Iranians at the time the study was conducted. This app allows easy sharing of information across various online forums. The stakeholders were given a brief summary of the aims of the exercise and the definition of the criteria in plain language, and were invited to rank the five criteria in a descending order based on their system of values. Sixty-eight stakeholders from the following groups participated: 16 patients with chronic diseases; 14 caregivers; 23 health care professionals (including 12 medical doctors, 6 nurses, 3 pharmacists, 1 dentist, and 1 midwife); 3 social workers; 9 social and environmental activists; and 3 pharmacists from industry.

The group of stakeholders collectively allocated the highest average score to the criterion *impact on health* (4.13). This was followed by *feasibility* (3.44), *impact on economy* (2.71), *equity* (2.56), and *capacity building* (2.19). To calculate the weight of each criterion, these average scores were divided by 3.00; ie, the average score in case of equal value of all the five criteria. Tables S6 and S7 in **Online Supplementary Document(Online Supplementary Document)** provide further information on stakeholders’ engagement.

**Table 2:** The top 10 priorities for health research for Iran, ranked by wRPS

Rank	Proposed RQ	wRPS	AEA	Feasibility	Impact on health	Impact on economy	Capacity building	Equity
**1**	How do we reform Iran's health insurance system to improve equity?	84.5	68	81	91	91	63	91
**2**	What should be Iran's non-communicable diseases prevention strategy? How could it be integrated into primary health care?	84.2	66	86	93	83	69	83
**3**	What are the most effective and cost-effective population-level interventions for reducing and managing non-communicable diseases (eg, ischemic heart diseases, diabetes, stroke, hypertension, and dementia) and road traffic injuries in the Iranian context?	84.2	65	84	91	88	64	88
**4**	How do we tailor our primary qualifications (eg, Doctor of Medicine, and Doctor of Pharmacy) and higher qualifications (eg, specialist training in psychiatry) in medical and health sciences in Iran to better serve the needs of the nation?	81.2	60	84	83	79	80	79
**5**	What are the leading non-communicable diseases (eg, diabetes, stroke, cardiovascular disease, hypertension, and cancer) in Iran now and in 2030? How are they distributed across the country and what are their underlying causes?	79.6	60	86	87	79	57	79
**6**	To what extent are health resources and services equally distributed across the country? To what extent do they meet the needs of the people? How do we reduce inequality in health service access in Iran and achieve Universal Health Coverage?	79.2	65	85	89	76	60	76
**7**	What is the health status and common health problems in Iran's elderly population? How will these change by the year 2030? What is the economic burden of these problems and how can we reduce this burden?	78.6	58	87	89	74	58	74
**8**	What is the current status of antibiotic resistance in Iran and future predictions? What is the current antibiotic prescription pattern(s) of medical practitioners in Iran? What can we do to promote more rational use of antibiotics?	78.1	53	85	88	77	51	77
**9**	What are the health impacts of Iran's water crisis? For example, how has the Lake Urmia water crisis impacted the health of residents in nearby provinces? What can the Ministry of Health and Medical Education and health centers across the country do to respond to water crises?	78.0	58	75	86	81	60	81
**10**	How can we replace the current physician-centered system with a team-based care approach (ie, the provision of health services by at least two health professionals including medical doctors, clinical pharmacists, nurses who work collaboratively with patients, family caregivers, and community service providers to achieve care)?	77.8	60	73	84	78	72	78

RQ – research question; wRPS – weighted research priority score; AEA – average expert agreement

The top 10 priorities comprised a wide range of RQs: 9 contained HPSR components with one RQ being focused on medical education; 6 entirely or partially fell into the scope of epidemiological research; and one addressed planetary health. The top priority RQ “How do we reform Iran's health insurance system to improve equity?” also scored highest for *impact on economy* (RPS = 90.7) and *equity* (RPS = 91). The second, the third, and the fifth priorities all addressed NCDs: two HPSR and one epidemiological question. They focused on NCD prevention strategy for Iran and its integration into the primary health care; seeking effective and cost-effective population-level interventions for reducing and managing NCDs and road traffic injuries; and an epidemiological assessment of NCDs and their underlying causes across Iran and making projections for 2030. Both of the HPSR questions that addressed NCDs have also scored highest for *impact on health*.

The 4^th^ priority focused on tailoring medical qualifications to better serve the needs of the nation, and it was agreed among the experts that this RQ would provide the greatest opportunity for *capacity building*. The experts collectively proposed that the 6^th^ priority, which focused on ways to achieve Universal Health Coverage (UHC), was identified as a rather feasible question to address (ranked 8^th^ for *feasibility*) and with a good potential for making an *impact on health* (ranked 4^th^). This priority was followed by proposing investigation of the current and the future common health problems in Iran’s elderly population and identifying strategies to reduce their economic burden. The consensus of experts suggested that this question is the most feasible among all the 128 RQs.

One planetary health question that was identified as a top-10 priority addressed health impacts of water crisis in Iran and how the health system should respond to it. Another priority suggesting a health systems research to replace physician-centered system with a team-based one was found to provide a great opportunity for *capacity building*. Another top-10 priority proposed assessing the status of antibiotic resistance in Iran and investigating ways to promote rational use of antibiotics.

In general, the level of agreement among the experts was between 67.5% and 34.58%. The RQs for which the greatest level of expert agreement was observed were generally those that received the highest RPS. Seven of the top-10 priorities had the greatest agreement scores, with the top-3 RQs having had received the highest AEA (65%, 66%, and 68%). On the other hand, RQs that had been the most controversial were those that ranked lower than the median based on the expert scoring (RPS<68.3).

Among the 10 RQs from the bottom of the ranking (wRPS range = 52.5-28.4), 8 addressed traditional Iranian medicine and Islamic-Iranian values, while two were very specific questions related to dementia. Examples of low-scoring RQs related to dementia were “How do behavioural and psychiatric symptoms of dementia patients differ between Iran and other countries?” (wRPS = 41.4); or “At which stages of dementia do people seek medical assistance in Iran, and how does this vary between groups with different levels of education?” (wRPS = 52.5). The RQ that received the lowest score (wRPS = 28.4) was: “How should we remember and honour the most influential medical scientists in Iran's history?”

## DISCUSSION

This exercise identified the research priorities that have a potential to assist Iran in addressing the knowledge gaps to achieve its long-term health targets as outlined in the National GHPs by 2025 and the SDGs by 2030. This was the first national-level health research priority-setting exercise in Iran that aggregated independent input from prominent Iranian researchers, policy makers and funders, and a group of stakeholders from the wider society. While the identified research priorities covered a diverse range of issues related to health, the majority of the proposed RQs, as well as 90% of the top-ranked questions, had aspects of HPSR. This is not surprising given the context of this study, which addressed a set of broad national health targets within a relatively short period (ie, the next 5 years) [11].

The HPSR questions that ranked among the top quartile of the 128 RQs predominantly related to financing, governance, and/or service delivery. This corresponds quite well with Iran’s ongoing Health Transformation Plan—launched in 2014—which aims to provide UHC by 2025, improve the quality of health care services, and achieve financial protection [17]. Furthermore, seeking ways to improve efficiency of the health system was reflected in several of the top-10 RQs, eg, reform of the insurance system; prevention and management of NCDs at different levels of the health system; and replacing the physician-centered health care system with a team-based one. This may suggest that, although Iran’s investment in health care has substantially increased since 2015 [17], the system has not yet succeeded in efficiently using its resources, either financial or human resources.

The priorities also clearly highlighted the significance of addressing questions that seek cost-effective population-level interventions and health system strategies to prevent NCDs and road traffic injuries in Iran. It was also agreed that studying the current and the estimated future distribution of NCDs and their underlying causes across Iran as well as the common health problems in Iran’s elderly population should be prioritized. Such results reflect the need for further investment in addressing the knowledge gaps in a country facing a rapidly increasing burden of NCDs [2] with an ageing population [18]. Such challenges reinforce the need for improving efficiency of the health system.

Consensus of the participants also indicated that Iran’s success in achieving its long-term health targets requires investment in studying the health impacts of environmental challenges, eg, water crisis. It had been argued previously that securing sustainable water resources in Iran requires immediate coordinated multi-sectorial action [19]. Another top-10 priority highlighted the importance of tailoring medical education to better serve the needs of the society. This question was raised for the primary qualification (eg, Doctor of Medicine) and was also specified for the curriculum of specialist training in psychiatry.

The questions related to the values embedded in the rich history of medical sciences in Iran were relatively prevalent among the originally proposed RQs (9 out of 128). However, the majority of them were ranked near the bottom of the list. This could suggest that, although a number of Iranian academics agreed that knowledge gaps regarding these issues should be addressed, it seems that it was difficult for these questions to meet the criteria of this exercise. It is likely that in a prioritization exercise within a different context, eg, considering a longer time scale, or using a different set of criteria, these questions could receive a higher average score.

It is worth mentioning that since 2000, Iran’s MOHME has been building capacity in medical universities across the country and in different departments of the Ministry to carry out research prioritization exercises [20]. Research priorities had been identified either at the national level in specific areas, eg, prevention of cervical cancer [21], HIV and AIDS [22], and patient safety [23], or at the institutional level, eg, in health systems research [24], or medical education [25]. However, none of these studies had engaged appropriate stakeholders from the wider society; the majority lacked a clearly-defined context; and all had used methods that included a panel of experts, rather than a structured and replicable approach.

Furthermore, while efforts to improve governance of health research in a developing country are admirable, it is unclear how the priorities were being set at the national level. It was reported in 2016 that a total of 6723 “research priorities” were collected from Iran’s medical universities [26], but this number is too large to consider each idea a “priority”. The report [26] continued that the collected research questions were grouped into nine “main areas”, but those were also too broad to be considered priorities that could direct national health research investments. A qualitative study investigated the barriers to evidence-based decision-making in Iran’s health system in 2012 [8]. The participants identified the following issues: (i) lack of a systematic prioritization mechanism; (ii) priorities being identified by MOHME and not being communicated with academics; and (iii) priorities being too general, thus failing to guide researchers.

Our study adapted the CHNRI method, which has several advantages (**Box 1**) [10-13]. Compared to previous national-level CHNRI exercises conducted in South Africa, India, Brazil, China, and one study that included Malawi, Nigeria, and Zimbabwe [11,27], this study had a relatively similar time scale, ie, 5-10 years. Perhaps this shows that it is of more interest for national-level studies to set the priorities with shorter periods in mind. This study had several strengths compared to previous CHNRI exercises. As the method has already been applied in over 50 exercises [11], plenty of published guidance was available to help adapt the method to our needs. The management group considered the modifications in the previous CHNRI studies [11] and this helped to easily reach a consensus over the five criteria most relevant to the present study. Furthermore, previous CHNRI exercises had a response rate between 30-70% [11], while in this exercise more than 70% of contacted participants responded both to a request to generate RQs and to score them. This shows that the exercise has been successful in engaging the experts with the process and the results are less likely to be biased. Moreover, the wide range of final research priority scores (84.5-28.5%) indicated a good level of discrimination between the RQs in meeting the five criteria. Finally, only 24% of previous CHNRI exercises had managed to engage an appropriate group of stakeholders in the process [14].

We explored the impact that the stakeholders’ assigned weights had on the final ranking of the RQs. Their input led to minor changes in the order of the questions, and also promoted two new RQs among the top-10 priorities, one addressing the common health problems in the elderly and another regarding antibiotic resistance. In the top quartile, notable examples of questions that were helped by the stakeholders' input were: (i) impact of climate change on air quality of Iranian cities and its health effects; (ii) community-based strategies to reduce energy consumption and environmental pollutants to improve health; and (iii) community-based interventions for reducing high-risk behaviors (intravenous drug use, unprotected sex) amongst adolescents living in deprived areas. In summary, the stakeholders’ input did not lead to a drastic change in the final ranks, but it helped several RQs with a focus on community participation to improve their ranks.

In terms of the level of agreement among experts, the AEA ranged between 67.5 and 34.5%. All of the RQs that were the most controversial were RQs that have ranked lower than the median RPS of 68.3. The greatest level of controversy seems to have been observed among the questions that were either too broad or too specific. This study had some apparent limitations, summarized in **Box 3**.

Box 3 **Limitations of this CHNRI exercise**

The findings of the CHNRI exercises may represent a biased opinion of a limited group of involved experts. In terms of the initially proposed RQs, some valid RQs will not be proposed and this is an inherent limitation. In terms of scoring, it has been shown that the collective opinion of the experts in CHNRI processes stabilizes with involving 45-50 participants [28]. Thus, the number of experts in this study (48 persons) was sufficient to produce robust results, which would be highly unlikely to change with adding further scorers. Regarding diversity of the sample, a considerable effort was made to include experts with a wide range of disciplinary backgrounds and views. Even so, more than 70% of the identified experts had a background in medical sciences. It appears that health research in Iran is very much dominated by medical scientists, even though some may have focused their research on areas that are not purely medical. For instance, the majority of the engaged experts in this exercise with academic degrees in medical sciences, had several years of experience in the multi-disciplinary field of HPSR. There does not seem to be a significant difference between the disciplinary backgrounds of the initial invitees, those who generated RQs, and/or the scorers, while the high response rate makes such a bias less likely.

The potential bias in this exercise could result from the process through which the experts were identified, because this relied on the input from three members of the steering committee (RM, ZA, and PM), with a snowballing for less represented fields. Following the standard CHNRI guidelines [29], one third of the experts were identified by searching for the most prolific researchers in the fields relevant to the health targets of this exercise. No time limit was considered in the search strategy. The experts were found among the most productive 50 authors in research areas relevant to the scope of this study through searching in Web of Science and Scopus. One author (PM) went through a random sample of the publications of the most productive authors to identify whether the papers were original research and could confirm that their authors were real experts in the field. Another applied approach was to identify people who have held scientific roles in Iranian scientific societies, universities, or executive organizations. Careful consideration was given to ensure that the invitees had a good understanding of research in their field, and were not solely holding executive roles.

In total, 11 invitees did not participate in generating RQs, nor in the scoring of RQs. Only one invitee declined participation due to concerns over the CHNRI methodology, questioning its ability to replace debate. Of the 50 experts who participated in generating RQs, but did not score them, only one declined for a specific reason, believing that the RQs needed a more comprehensive assessment rather than numerical scores against a set of criteria. The most likely reason for others to decline was time constraint, ie, failing to meet the deadline for responding.

A review of 50 CHNRI studies has found that the redundancy rate in the initial list of proposed RQs is nearly over 50% [11]. In this study it was almost the same: a total of 251 RQs was reduced to 128. During this step, some of the initially proposed descriptive RQs were merged with interventional RQs that addressed the same health problem, and this could have led to biased responses towards one part of the question. Another issue worth mentioning was that, although the compiled list of RQs was refined and revised multiple times by the steering committee before sending out to the scorers, there were still RQs that had some degree of overlap, which was very difficult to avoid completely. Finally, it should be noted that all RQs were initially proposed in Persian, then translated into English (by PM) to be discussed in the steering committee, and then the compiled list of 128 RQs was back-translated into Persian. Within this process, very careful consideration was taken for translations being well-representative of the original meaning.

The identified priorities seem plausible within the context of the study, which involves a country with an ageing population, facing a high burden of NCDs and road traffic injuries while in parts still struggling with endemics of infectious diseases, experiencing accelerated environmental changes, having a large number of students at universities of medical sciences, and undergoing a Health Transformation Plan to achieve UHC. On the other hand, we should analyse why the majority of questions addressing mental health, substance abuse, or dementia did not score highly despite a high burden of disability and death which they cause in Iran [4]. It is possible that first, the way in which the questions in those areas were framed did not seem *feasible* enough to the scorers. Second, the low scores that these questions have received for *impact on economy* may suggest insufficient awareness of the majority of the engaged experts about the burden that non-physical health conditions could cause. It is also worth mentioning that while the consensus of experts in this exercise strongly supported the need for investment in further studies on NCDs, one of the top-10 priorities addressed antimicrobial resistance. This highlighted that although communicable diseases are contributing to less DALYs in Iran, they continue to remain important. Diseases such as influenza, brucellosis, and tuberculosis are still major endemics in Iran [3], and the country’s capacity in studying and controlling outbreaks of neglected, emerging, and/or re-emerging infectious diseases requires improvements [3].

The identified priorities described in this paper guide research that is likely to be feasible to be conducted within the next five years, and bear a higher impact on health and economy and lead to capacity building and equity. The authors hope that this paper will first contribute to the field of health research priority-setting as one of the most recent applications of the CHNRI method at a national level. Second, this was the first application of the CHNRI method in Iran and we hope that the findings of this study will be utilized in making funding decisions within the next five years. Advocacy of the findings could be done through holding meetings with decision-makers at different departments of MOHME, the Islamic Parliament Research Center, Academy of Medical Sciences, and research departments of major Iranian universities of medical sciences. Summaries of the results should become available online for open and easy access of all researchers and stakeholders. We also encourage different funding agencies to compute the weights for the criteria using the input from their own major stakeholders to adjust the priorities with their organizations’ targets.

Iran has got a substantial health research capacity [6] and proved interest in prioritizing research [20]. Now that the CHNRI method has been successfully introduced in Iran, we recommend its application to identify the research priorities in more specific domains, eg, for certain health conditions with a high burden in Iran, or for specific target populations. It is also recommended to conduct the CHNRI studies at a sub-national level. This would assist with identifying the priorities based on the needs of each region of this relatively large and populous country, where each part deals with particular health challenges ranging from water scarcity and air pollution to substance abuse or challenges imposed by sharing borders with countries in conflict. The development of a Massive Open Online Course (MOOC) for the Iranian audience could facilitate implementation of the method at a larger scale. We also recommend this national-level exercise to be repeated every five years to be aligned with Iran’s 5-Year Development Plans. Within the next five years, we will be tracking research grant calls and application to monitor if the findings of this study are implemented, and will highlight the priorities that will not be addressed. Finally, this exercise was the first implication of the CHNRI method in the World Health Organization (WHO) Eastern Mediterranean Region (EMR). The research team will pursue efforts to share the results with the WHO EMR Office that may assist conduct of the exercise in countries with a fairly similar context to Iran.

## CONCLUSION

The main messages of this study were that addressing equity by reforming insurance system and improving equality in health services access should be prioritized in Iran. Furthermore, research on preventive strategies for the leading NCDs and road traffic injuries are needed, as well as further epidemiological research on NCDs and the health problems of the elderly population. This should happen in parallel to a tailored curriculum of qualifications in the universities of medical sciences, where a team-based health care system should be promoted. Research is also needed on over-/mis-use of antibiotics and the health impacts of water crisis.

This study was a good example of a national-level health research priority-setting process and a rare example where a national research funding body used the process to decide on health research funding in a holistic way. This helped to overcome problems of previous research priority-setting exercises, with further implementation now under way in Iran. This exercise also provides a model to public research funding bodies in other countries that could be adapted to improve decision-making on funding allocation for health research, especially in low resource settings.
