# Supplementary material for: Setting research priorities to achieve long-term health targets in Iran
Source: J Glob Health. 2018 Oct 13;8(2):020702. doi: 10.7189/jogh.08.020702 (PMC6188089; doi:10.7189/jogh.08.020702)
Supplement: Online Supplementary Document [file jogh-08-020702-s001.zip › jogh-08-020702 Supp Doc 2.pdf]

Supplementary file to the manuscript entitled "Setting Research Priorities to Achieve Long-Term Health Targets in Iran"

Supplementary Table S1: Defining the context of Iran's CHNRI health research priority-setting exercise

Defining the context

In the first step, a steering committee was formed for running the Child Health and Nutrition Research Initiative (CHNRI) exercise in Iran. Parisa Mansoori (PM) as the coordinator of the steering committee invited four people to sit on the committee as follows: (1) Professor Igor Rudan (IR); (2) Dr Kit Yee Chan (KYC); (3) Professor Reza Majdzadeh (RM); and (4) Dr Zhaleh Abdi (ZA). IR and KYC were invited for their expertise in the CHNRI methodology and extensive experience in its implementation in multiple national and global exercises. RM and ZA were invited for ensuring that the CHNRI method was adapted properly within the context of Iran. They also facilitated identification of and access to the study participants.

Through several meetings and discussions within the steering committee, different components of the context of the exercise were defined, including, (i) health targets on which the exercise focused and the agendas where the targets are outlined (i.e. Iran's long-term health targets as outlined in Iran's General Health Policies and in the United Nations' Sustainable Development Goals); (ii) geographic limits (i.e. national); and (iii) the time scale within which the research is expected to be funded and conducted (i.e. the next 5 years). In terms of the time scale of the exercise, a short-term period was aimed, because most of the national plans in Iran are made for 5-year periods. Furthermore, the majority of research funding bodies in Iran are run by the government, thus their funding policies often change every 4 to 8 years with the change of the President of the country and/or his Cabinet.

In terms of the long-term health targets, Iran has two major commitments for improving population health. The first commitment (i.e. Iran's national commitment) are the General Health Policies (GHPs) by 2025 and the second one (i.e. Iran's international commitment) is to deliver the United Nations' Sustainable Development Goals (SDGs) by 2030. Aligning the national and international commitments facilitates the monitoring and evaluation of the progress towards achieving the long-term targets. Therefore, this exercise included the health targets of Iran as outlined in both its national and international major commitments. A full list of the health targets that were considered in this CHNRI exercise are provided in **Table S2**.

In terms of the international agenda, the SDGs were included in this exercise. The SDGs are a set of interconnected goals that build on the successes of the Millennium Development Goals, while introducing new areas, such as climate change, economic inequality, and sustainable consumption. The SDGs were adopted by the UN General Assembly in 2015, and Iran is one of its signatories. Regarding Iran's national agenda, the GHPs, i.e. a set of 14 long-term health targets, are included. The GHPs were notified in 2014 by Iran's Supreme Leader—Ayatollah Seyed Ali Khamenei—who according to the Islamic Republic of Iran's constitution, is responsible for outlining the general policies for the country. The GHPs aim to provide Iran with a framework to achieve the health-related goals of Iran's Vision 2025. The Vision 2025 is a 20-Year national strategic plan that was released in 2004 and outlines political, economic, and social goals, which achieving them presumably could facilitate Iran's broader ambition, i.e. "becoming the Middle East's top power by 2025".

Reviewing the content of the GHPs and the health targets of the SDGs revealed a high degree of overlap between the targets across the two agendas. Among the 17 goals that are proposed in the SDGs, Goal 3 specifically addresses health issues and is described as a goal for "ensuring healthy lives and promoting the well-being of all at all ages" [1]. In addition to the 13 targets outlined under this goal (i.e. 3.1-3.9, and 3.a; 3.b; 3.c; and 3.d), 8 additional SDG health targets were included from goals other than Goal 3. This included the targets that were proposed in the 2016 report of the World Health Organization on Monitoring Health for the SDGs [2]. Five of the GHPs are not covered by the SDGs as they are quite specific to the context of Iran (including GHP 1, GHP 2, GHP 12, GHP13, and GHP 14)

Supplementary Table S2: List of the health targets in Iran's health research CHNRI priority-setting exercise

Included health targets

General Health Policies of the Islamic Republic of Iran (GHPs)

GHP 1: Providing health services (including medical and health sciences education and research services, and preventive, curative, and rehabilitative care services) based on human and Islamic principles; and institutionalising such principles in the society as follows:
Improving the system of recruitment, assessment, teaching, and training of the students, faculty members, and directors in scientific and academic environments according to the Islamic, professional and medical codes of ethics
GHP 1.1: Raising awareness in the public about their [health-related] social rights and responsibilities and use the capacity of the health-care centers to promote spirituality and Islamic ethics in the society
GHP 2: Fulfilling Universal Health Coverage (UHC) in all the laws, regulations, and policies through:
GHP 2.1: Prioritizing prevention over treatment
GHP 2.2: Updating national medical and health plans
GHP 2.3: Reducing environmental health hazards and the relevant risk factors based on credible scientific evidence
GHP 2.4: Preparing "Health Attachments" for the national development projects ["Health Attachment" was a term proposed for the first time in the 5th National Development Plan of Iran—in Section B of Article 32—stating that the national development projects should have an attachment that would discuss the potential health impacts of the project]
GHP 2.5: Improving the health indexes for becoming the first country of the Southwest Asia
GHP 2.6: Improving and complementing the current assessment and monitoring systems to protect [health-related] rights of the patients and the nation and to ensure the proper implementation of the GHPs
GHP 3: Improving mental health of the nation by: promoting the Islamic-Iranian lifestyle; strengthening family foundation; removing factors that cause tension in individual and social life of the people; promoting moral and spiritual teachings; and improving mental health determinants
GHP 4: Establishing and strengthening the required infrastructure for manufacturing pharmaceutical active ingredients and products, vaccines, biologic drugs, and medical devices and equipment that meet the international quality standards
GHP 5: (i) Inhibiting induced demand; (ii) Inhibiting prescription of medications that are outside the clinical guidelines and the Generic National Pharmaceutical System of Iran; (iii) Policymaking and efficient oversight on manufacturing, use, import of pharmaceuticals, vaccines, biologic drugs, and medical devices aiming to support domestic production and increasing national capacity for export
GHP 6: Supplying food security; and equitably providing healthy and adequate food, clean air and water, public sports facilities, and safe sanitary products that are all approved by the national, regional, and international standard criteria
GHP 7: Sharing the duties regarding stewardship, financial supplying, and providing health services with the aim of responding to the needs, achieving equity, and providing appropriate medical services to the community as follows:
GHP 7.1: Stewardship of the health system by the Ministry of Health and Medical Education (MOHME), including policymaking, designing and implementing plans, designing strategies, evaluation, and oversight of the plans
GHP 7.2: Management of resources in the health sector through the insurance system with the central role of MOHME and the cooperation of other organizations and entities

GHP 7.3: Providing services by resources from the government [e.g. the ministries], the public resources [e.g. the municipalities] and the private sector [e.g. charities]

GHP 7.4: Organising the abovementioned tasks based on the mechanisms that are determined by law

GHP 8: Increasing and improving the quality and the safety of the [health] services and developing a comprehensive and integrative health-care [system] that focuses on equity and is responsive [to the nation's needs] and emphasises transparent informing, efficacy, efficiency, and productivity within the Primary Health-Care (PHC) and is in line with the referral system by:

GHP 8.1: Promoting decision-making and operation based on scientific evidence; and codifying standards and guidelines; undertaking Health Technology Assessment; establishing the referral system by prioritizing prevention and health improvement and integrating them with the medical education system

GHP 8.2: Increasing the quality and the safety of health-care services by promoting clinical authority and determining standards

GHP 8.3: Codifying a comprehensive health-care plan to support disabled people and disabled Iranian veterans aiming to improve their health status and to empower them

GHP 9: Improving the quantity [the number of people who are covered] and the quality of health and medical insurance aim to:

GHP 9.1: Achieving universal public health insurance

GHP 9.2: Achieving full coverage of basic medical needs by insurance companies for all to reduce the patients' share in medical expenses

GHP 9.3: Providing services beyond the basic insurance by complementary insurance that follows transparent regulatory frameworks

GHP 9.4: Preparing packages of comprehensive health and medical services at the levels of basic and complementary insurance by MOHME

GHP 9.5: Strengthening a competitive market for medical insurance companies

GHP 9.6: Codifying tariff policies based on evidence

GHP 9.7: Modifying the payment models, in a way that it would be based on the quality of services, be more efficient, and would pay special attention to health promotion activities and to prevention in deprived areas

GHP 10: Sustainable health financing

GHP 10.1: Making the amount of expenses, activities ,and earnings [within the health system] transparent

GHP 10.2: Increasing the proportion of the Gross Domestic Product (GDP) and the public budget that is invested in health. In this way Iran's investment in [health-care] should exceed the average investment of the countries in the region [The Middle East], thus it would fulfil the targets as set in the Vision policy [Iran's 20-year national strategic plan by 2025].

GHP 10.3: Adding taxes on products that can be harmful to health

GHP 10.4: Improving the equitable distribution of government health-care subsidies

GHP 11: Raising awareness, responsibility, capability, and systematic and active participation of individuals, families, and the community in supplying, maintaining, and improving health by using the capacity of different cultural and educational entities as well as the media, under the supervision of MOHME.

GHP 12: Studying, understanding, developing, promoting, and institutionalising traditional Iranian medicine

GHP 12.1: Prompting cultivation of medicinal plants under the supervision of the Ministry of Agriculture Jihad and supporting the development of technical and scientific innovations in manufacturing traditional medicines under the supervision of MOHME

GHP 12.2: Standardising and updating the diagnostic and curative methods that are recommended in traditional Iranian medicine
GHP 12.3: Exchanging knowledge with other countries regarding traditional medicine
GHP 12.4: MOHME supervising the provision of health services and products that use traditional Iranian medicine
GHP 12.5: Establishing links between traditional Iranian medicine and modern medicine
GHP 12.6: Modifying people's lifestyle, particularly their diet [by using traditional Iranian medicine]
GHP 13: Improving the quantity [increasing the capacity] and the quality of the medical education system [in Iran] to make it become targeted, health-oriented, and based on the needs of the nation; and efficiently training human resources to respond to the needs in an equitable manner, and [training people in a way that they] would be committed to the Islamic and professional ethics and would have essential skills to respond to population health needs across different parts of the country
GHP 14: Revolutionising medical research [medical research system in Iran] strategically with an approach to [promote] innovation system and planning for making Iran a pioneer in science, technology, and in the provision of medical services and making Iran to become a medical hub in Southwest Asia and in the Islamic world

---

Health-related targets of the United Nations' Sustainable Development Goals (SDGs)

SDG (3.1) By 2030, reduce the global maternal mortality ratio to less than 70 per 100,000 live births

SDG (3.2) By 2030, end preventable deaths of newborns and children under 5 years of age, with all countries aiming to reduce neonatal mortality to at least as low as 12 per 1000 live births and under-5 mortality to at least as low as 25 per 1,000 live births

SDG (3.3) By 2030, end the epidemics of AIDS, tuberculosis, malaria and neglected tropical diseases and combat hepatitis, water-borne diseases and other communicable diseases.

SDG (3.4) By 2030, reduce by one third premature mortality from non-communicable diseases through prevention and treatment and promote mental health and well-being.

SDG (3.5) Strengthen the prevention and treatment of substance abuse, including narcotic drug abuse and harmful use of alcohol.

SDG (3.6) By 2020, halve the number of global deaths and injuries from road traffic accidents.

SDG (3.7) By 2030, ensure universal access to sexual and reproductive health-care services, including for family planning, information and education, and the integration of reproductive health into national strategies and programmes.

SDG (3.8) Achieve universal health coverage, including financial risk protection, access to quality essential health-care services and access to safe, effective, quality and affordable essential medicines and vaccines for all.

SDG (3.9) By 2030, substantially reduce the number of deaths and illnesses from hazardous chemicals and air, water and soil pollution, and contamination.

SDG (3.a) Strengthen the implementation of the WHO Framework Convention on Tobacco Control in all countries (including controlling production and consumption)

SDG (3.b) Support the research and development of vaccines and medicines for the communicable and non-communicable diseases that primarily affect developing countries, provide access to affordable essential medicines and vaccines, in accordance with the Doha Declaration on the TRIPS Agreement and Public Health, which affirms the right of developing countries to use to the full the provisions in the Agreement on Trade-Related Aspects of Intellectual Property Rights regarding flexibilities to protect public health, and, in particular, provide access to medicines for all.

SDG (3.c) Substantially increase health financing and the recruitment, development, training and retention of the health workforce in developing countries

SDG (3.d) Strengthen the capacity of all countries, in particular developing countries, for early warning, risk reduction and management of national and global health risks.

SDG (2.2) By 2030 end all forms of malnutrition, including achieving by 2025 the internationally agreed targets on stunting and wasting in children under five years of age, and address the nutritional needs of adolescent girls, pregnant and lactating women, and older persons.

SDG (6.1) By 2030, achieve universal and equitable access to safe and affordable drinking water for all

SDG (6.2) By 2030, achieve access to adequate and equitable sanitation and hygiene for all and end open defecation, paying special attention to the needs of women and girls and those in vulnerable situations

SDG (7.1) By 2030, ensure universal access to affordable, reliable and modern energy services (Clean household energy to use for cooking, heating and light)

SDG (11.6) By 2030, reduce the adverse per capita environmental impact of cities, including by paying special attention to air quality and municipal and other waste management

SDG (13.1) Strengthen resilience and adaptive capacity to climate-related hazards and natural disasters in all countries

SDG (16.1) Significantly reduce all forms of violence (i.e. physical, mental, or sexual violence) and related death rates and conflict-related deaths

SDG (16.2) End abuse, exploitation, trafficking and all forms of violence against and torture of children

Supplementary Table S3: Overview of the process of selecting and defining the criteria

**Selecting and defining the criteria**
The CHNRI method introduces a transparent set of criteria that could discriminate between many competing research options. CHNRI's "standard" criteria include (i) answerability, (ii) effectiveness, (iii) deliverability, (iv) potential for a substantial reduction of disease burden, and (v) impact on equity [3]. However, the CHNRI method provides flexibility to modify the criteria according to the context [3]. Using this flexibility, the selected criteria and their definitions were modified for this exercise, based on the input from a management group. The management group comprised the steering committee members (including PM, IR, KYC, RM, and ZA) and four other people who held positions that allowed them to influence Iran's major health research policy making and/or funding decisions.<br><br>On the first step, the steering committee discussed the suitability of the "standard" CHNRI criteria and the other criteria that have been used in previous CHNRI exercises for the context of this exercise. Two new criteria were proposed as they seemed to fit within the context of Iran. After several discussions where each of the members of the steering committee argued for inclusion and/or exclusion of any of the criteria, a consensus on a shortlist of 9 criteria and a definition for each was achieved. The shortlist included the following: (i) feasibility; (ii) impact on health; (iii) impact on economy; (iv) capacity building; (v) equity; (vi) impact on Iran's scientific rank; (vii) potential for translation; (viii) impact on creating wealth; and (ix) long-term impact.<br><br>On the second step, the other four members of the management group were invited to independently rank the nine criteria, choose the top-five, and provide comments that could improve the proposed definitions of the criteria. These participants were invited from the Academy of Medical Sciences (Professor Fereidoun Azizi); Department of Research and Technology of MOHME (Professor Shahin Akhondzadeh); Department of Curative Affaires of MOHME (Dr Jafar Sadegh Tabrizi); and Department of Research and Technology of Tehran University of Medical Sciences (Professor Masud Yunesian). Except for the first invitee who represented the Academy of Medical Sciences, the other three chose to express their personal opinion.<br><br>On the third step, each of the steering committee members ranked all the nine criteria too. By counting the total votes for each criterion as well as counting the scores that each criterion had received (based on the ranking system), the top-five criteria were selected as follows: (i) feasibility; (ii) impact on health; (iii) impact on economy; (iv) capacity building; and (v) equity. The top-five criteria turned out to be the same using both approaches (counting the votes or counting the value based on ranking). Finally, by summarising the comments from all, PM revised the definitions provided for each criterion. Dr Bahareh Yazdizadeh from the Knowledge Utilization Research Center at Tehran University of Medical Sciences provided intellectual contribution to improving the definitions.

Supplementary Table S4: Identification, invitation, and participation of the experts in Iran's CHNRI health research priority-setting exercise

Identification, invitation, and participation of the experts

In total, we identified 70 experts—in the fields relevant to the health targets that were included in this exercise—across a reasonably wide range of disciplinary backgrounds and views. To identify experts, the Web of Science and Scopus databases were searched for the most prolific researchers in Iran in the areas related to the included health targets in this study. To do so, PM went through a random sample of the publications of the most productive authors to identify whether the papers were original research and could confirm that their authors were real experts in the field.

Another approach was a systematic search of the web in Persian for experts who have held leading academic roles in Iranian scientific societies and or universities, or have held scientific roles in executive organizations, e.g. MOHME. Careful consideration was given to ensure that the invitees had a good understanding of research in their field, and were not solely holding executive roles. The final list of experts were checked and overseen by RM and ZA to ensure that the list had a comprehensive coverage of experts.

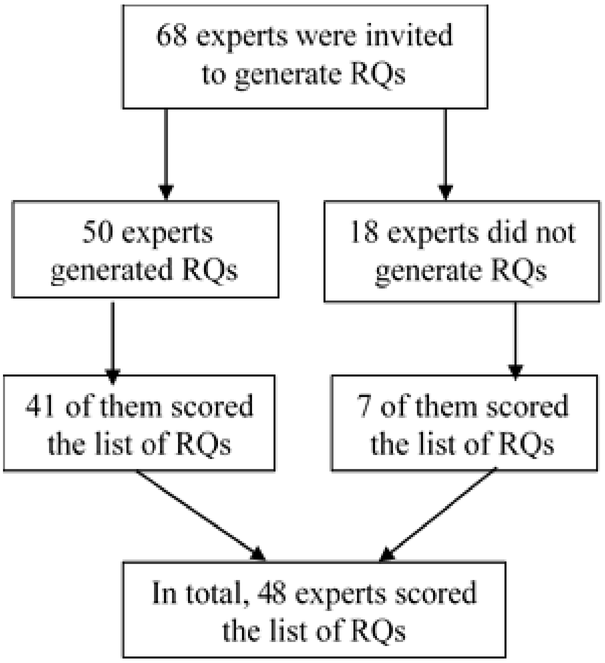

Figure S1. Experts' participation in the exercise

To diversify the range of expertise of identified researchers, which was predominantly from medical sciences backgrounds, snowballing was done by asking the identified experts in less represented areas (e.g. in social sciences, humanities, environmental sciences) to nominate other experts in their field. Despite such efforts, education background of the majority of the invitees (70%) was biomedical and/or public health sciences. This figure for those who contributed to generating RQs and to the scoring of the RQs was 74% and 75%, respectively.

Contact information of the identified experts was sought through combined searches in PubMed, Google, and in accessible contact and mailing lists. Of the 70 identified experts, updated contact information for two was not found (one anthropologist and one lawyer who were both based outside Iran). Therefore, eventually, 68 experts were invited to the exercise. Figure S1 summarises how many experts participated at each stage of the exercise.

On the scoring step, individualized scoring sheets that each researcher was later invited to fill out independently were prepared in Microsoft Excel. Each scoring sheet listed all the 128 RQs against the 5 criteria. The RQs were sorted randomly on each scoring sheet so that all of the RQs would have an equal chance of standing on higher or lower rows on the list. After sending out the scoring e-mails, the researchers were initially given 2 weeks to send back their responses. After 2 weeks, PM followed them up on a weekly basis and the majority responded within the following 2-3 weeks. To the researchers who wanted to decline participation due to time constraint, an option of scoring RQs against fewer criteria—instead of all the 5 criteria—was given. Of this group of researchers, only one of them accepted this offer and scored the RQs for only one criterion, and the rest of invitees who initially wanted to decline scoring due to time constraint scored the RQs against all the 5 criteria. One of the scorers argued that it was only possible to score the RQs for "Feasiblity", therefore he was encouraged to do so. In sum, 46 of the experts scored the RQs against the 5 criteria and two experts scored the RQs only for "feasibility".

Supplementary Table S5: Overview of the experts' background, participation, and responses

Number	Full Name	Discipline & Specialty	Reason for Decline
**Experts who generated RQs* and contributed to the scoring and are listed as co-authors of the paper (Two experts were anonymised upon their request)**			
**1**	Anonymous	Medicine	
**2**	Anonymous	Medicine, Social Medicine	
**3**	Aarabi, Mohsen	Medicine, Epidemiology	
**4**	Ahmadnezhad, Elham	Medicine, Epidemiology	
**5**	Ahmadnia, Shirin	Sociology, Sociology and Health	
**6**	Akhondzadeh, Shahin	Pharmacy, Neuroscience	
**7**	Azin, Ali	Medicine, Social Medicine and Sex Therapy	
**8**	Azizi, Fereidoun	Medicine, Endocrinology	
**9**	Dehnavieh, Reza	Management	
**10**	Eini-Zinab, Hassan	Sociology, Community Nutrition	
**11**	Farzadfar, Farshad	Medicine, Public Health	
**12**	Farzaei, Mohammad Hosein	Pharmacy, Traditional Medicine	
**13**	Ghanei, Mostafa	Medicine, Pulmonology	
**14**	Haghdoost, AliAkbar	Medicine, Epidemiology	
**15**	Heydari, Gholamreza	Medicine, Epidemiology	
**16**	Joulaei, Hassan	Pharmacy, Health Policy	
**17**	Kelishadi, Roya	Medicine, Paediatrics	
**18**	Khosravi, Ardeshir	Management	
**19**	Mahvi, Amir Hossein	Environmental Engineering	
**20**	Majdzadeh, Reza	Veterinary Medicine, Epidemiology	
**21**	Massah Bavani, Ali Reza	Environmental Studies, Climate Change	
**22**	Mesdaghinia, Alireza	Environmental engineering, Public Health	
**23**	Montazeri, Ali	Medicine, Public Health	
**24**	Mostafavi, Ehsan	Medicine, Epidemiology	
**25**	Motevalian, Seyed Abbas	Medicine, Epidemiology	
**26**	Nikfar, Shekoufeh	Pharmacy, Pharmacoeconomics and Pharmaceutical Management	
**27**	Nojoumi, Seyed Ali	Microbiology, Innovation	
**28**	Noroozian, Maryam	Geriatric Medicine, Cognitive Neurology	
**29**	Olyaeemanesh, Alireza	Medicine, Health Policy	
**30**	Omidvar, Nasrin	Community Nutrition	
**31**	Ostadtaghizadeh, Abbas	Medicine, Disaster	
**32**	Pourmalek, Farshad	Medicine, Public Health	
**33**	Rahimi, Roja	Pharmacy, Traditional Medicine	
**34**	Rahimi-Movaghar, Afarin	Medicine, Psychiatry	
**35**	Razaghi, Emran	Medicine, Psychiatry	
**36**	Sadeghi-Bazargani, Homayoun	Medicine, Epidemiology	
**37**	Soori, Hamid	Medicine, Epidemiology	
**38**	Tabrizi, Jafar Sadegh	Medicine, Epidemiology	
**39**	Vedadhir, AbouAli	Anthropology and Health Studies	
**40**	Yunesian, Masud	Medicine, Environmental Health	
**41**	Zare, Mehdi	Seismology	

Experts who only generated RQs (The anonymised experts either have not replied to the e-mail asking for their permission to be acknowledged, or have not provided permission)			
**42**	Kebriaeezadeh, Abbas	Pharmacy, Pharmacology and Toxicology	He did not reply to the scoring invitation.
**43**	Malekafzali, Hossein	Medicine, Epidemiology	He did not reply to the scoring invitation.
**44**	Pourreza, Abolghasem	Economics	He did not reply to the scoring invitation.
**45**	Anonymous	Medicine, Hepatology, Health Policy	He did not reply to the scoring invitation.
**46**	Anonymous	Medicine, Public Health, Disaster	Declined to score due to time constraint. He was offered to score the RQs for fewer criteria (instead of all the 5) but he did not reply.
**47**	Anonymous	Sociology	He decided not to participate in the scoring.
**48**	Anonymous	Psychology	He declined to score, believing that the RQs needed a more comprehensive assessment rather than numerical scores against a set of criteria
**49**	Anonymous	Medicine, Infectious Diseases	She did not reply to the scoring invitation.
**50**	Anonymous	Pharmacy, Traditional Medicine	He did not reply to the scoring invitation.
**Experts who only contributed to the scoring and are listed as co-authors of the paper**			
**51**	Hantoushzadeh, Sedigheh	Medicine, Gynaecology, Perinatal Fellow	For the first stage, she declined due to time constraint.
**52**	Kalantari, Naser	Medicine, Paediatrics	He did not reply to the first invitation.
**53**	Larijani, Bagher	Medicine, Endocrinology	He did not reply to the first invitation.
**54**	Mokri, Azarakhsh	Medicine, Psychiatry	For the first stage, he declined due to time constraint.
**55**	Naddafi, Kazem	Environmental Health Engineering	For the first stage, he declined due to time constraint.
**56**	Rashidian, Arash	Medicine, Health Policy	For the first stage, he declined due to time constraint.
**57**	Salehi Zalani, Gholamhosain	Management	He did not reply to the first invitation.
**Experts who did not participate after being contacted multiple times**			
**58**	Anonymous	Medicine, Paediatrics	For both stages, he expressed interest in participation although eventually did not reply.
**59**	Anonymous	Economics	After being invited to the first stage, he shared his views about the exercise and expressed interest in participation although eventually did not reply.
**60**	Anonymous	Medicine, Public Health	For the first stage, he said that he had already participated in an exercise using the CHNRI method and had found the exercise impossible; from a practical perspective where there is not enough details provided about each RQ and also from a conceptual perspective, which he always wondered whether the process could ever replace debate and judgement. For the second stage, he initially said he would give it a try although eventually did not reply.
**61**	Anonymous	Sociology, Demography	For the first stage, he declined to participate due to time constraint. Although he expressed interest in participating in the scoring, eventually did not reply.

**62**	Anonymous	Medicine, Infectious Diseases	For both stages, he expressed interest in participation although eventually did not reply.
**63**	Anonymous	Medicine, Surgery	For the first stage, he stated that he would be considering participation although eventually he did not reply. He did not reply to the scoring invitation.
**64**	Anonymous	Nursing, Disaster	For the first stage, he showed interest in participation although eventually did not reply. For the second stage, he did not reply.
**65**	Anonymous	Medicine, Pulmonology and Internal Medicine	Did not reply.
**66**	Anonymous	Medicine, Midwifery	For the first stage, she declined due to time constraint. For the second stage, she initially expressed interest in participation, but eventually did not reply.
**67**	Anonymous	Sociology, Community Nutrition	Did not reply.
**68**	Anonymous	Civil Engineering	For the first stage, he did not reply.<br>For the second stage, he declined participation due to time constraint.

*RQs: Research Questions
- The names within each of the four sections of the table are ordered alphabetically

Supplementary Table S6: Stakeholders' individual assigned scores to the criteria						
Criteria		Feasibility	Impact on Health	Impact on Economy	Capacity Building	Equity
Number	"5" entails the highest importance while "1" entails the least importance					
Health-care professionals (23 participants)						
Medical doctors (12 participants)						
1	Anonymous (Surgeon)	1	3	4	5	2
2	Anonymous (Geriatrician)	4	5	2	3	1
3	Anonymous (Psychiatrist)	5	4	1	2	3
4	Anonymous (Gynaecologist)	5	4	2	1	3
5	Anonymous (Neurosurgeon)	4	5	2	3	1
6	Anonymous (Surgeon)	4	5	1	2	3
7	Anonymous (Cardiologist)	5	4	2	3	1
8	Anonymous (Neonatal specialist)	4	5	3	2	1
9	Anonymous (Nephrologist)	5	4	3	1	2
10	Anonymous (Emergency medical doctor)	4	5	3	1	2
11	Anonymous (General Practitioner)	3	4	5	2	1
12	Anonymous (General Practitioner)	2	4	3	5	1
Dentist (1 participant)						
13	Anonymous (Dentist)	3	2	1	4	5
Nurse/Midwife (7 participants)						
14	Anonymous(Psychiatry nurse)	4	3	1	5	2
15	Anonymous (Neonatal Intensive care unit nurse)	1	4	3	2	5
16	Anonymous(Nurse at Spinal injuries unit)	5	4	3	1	2
17	Anonymous (specialty not identified)	2	5	4	1	3
18	Anonymous (retired nurse-specialty not identified)	5	4	1	2	3
19	Anonymous (Midwife)	5	4	3	2	1
20	Anonymous (Nurse in surgery unit)	5	3	1	2	4
Pharmacist in pharmacy (3 participants)						
21	Anonymous	4	5	3	3	2
22	Anonymous	1	5	3	2	4
23	Anonymous	5	4	3	1	2
Social worker, working in the area of addiction (3 participants)						
24	Anonymous	5	3	2	4	1
25	Anonymous	5	3	2	4	1
26	Anonymous	5	3	2	4	1
Patients with chronic conditions (16 participants)						
27	Anonymous (Thalassemia)	4	5	2	1	3
28	Anonymous (Cardiovascular diseases)	2	4	1	3	5
29	Anonymous (Thalassemia)	2	5	4	3	1
30	Anonymous (Cancer)	4	5	1	2	3
31	Anonymous (Cancer)	5	3	2	1	4
32	Anonymous (Cancer)	4	5	2	1	3
33	Anonymous (Congenital anomaly)	4	3	1	5	2
34	Anonymous (Obsessive Compulsive Disorder and sleep disorder)	5	3	4	1	2
35	Anonymous (Multiple Sclerosis)	5	4	3	2	1
36	Anonymous (Lower back pain)	1	5	4	2	3
37	Anonymous (not specified)	2	4	1	3	5
38	Anonymous (not specified)	1	5	3	2	4
39	Anonymous (Spinal injury)	4	5	1	2	3
40	Anonymous (Cancer)	1	3	5	2	4
41	Anonymous (Wilson disease / Liver transplant)	5	4	2	1	3
42	Anonymous (Rheumatoid Arthritis)	3	5	4	1	2
Caregivers (14 participants)						
43	Anonymous	2	4	1	3	5
44	Anonymous	1	5	4	2	3

45	Anonymous	5	4	3	1	2
46	Anonymous	5	4	2	3	1
47	Anonymous	1	5	3	2	4
48	Anonymous	1	3	4	2	5
49	Anonymous	4	5	2	3	1
50	Anonymous	5	4	2	3	1
51	Anonymous	2	5	4	1	3
52	Anonymous	4	5	3	1	2
53	Anonymous	4	5	2	1	3
54	Anonymous	3	5	4	1	2
55	Anonymous	1	4	3	2	5
56	Anonymous	3	4	5	2	1
**Pharmacists representing industry (3 participants)**						
57	Anonymous	5	4	3	1	2
58	Anonymous	5	1	4	3	2
59	Anonymous	5	3	4	2	1
**Social/Environmental activist (9 participants)**						
60	Anonymous	3	5	4	2	1
61	Anonymous	1	4	2	3	5
62	Anonymous	5	4	2	1	3
63	Anonymous	4	5	3	2	1
64	Anonymous	4	3	5	1	2
65	Anonymous	2	5	3	1	4
66	Anonymous	4	5	1	2	3
67	Anonymous	1	4	5	3	2
68	Anonymous	1	4	3	2	5

Supplementary Table S7: Stakeholders' input calculation

Criteria	Mean score (1-5)	Mean suggested weight
Feasibility	3.44	1.15
Impact on Health	4.13	1.38
Impact on Economy	2.71	0.90
Capacity Building	2.19	0.73
Equity	2.56	0.85

References

1. GBD 2016 SDG Collaborators. Measuring progress and projecting attainment on the basis of past trends of the health-related Sustainable Development Goals in 188 countries: an analysis from the Global Burden of Disease Study 2016. Lancet. 2017;390:1423-59.
2. World Health Organization. World health statistics 2016: monitoring health for the SDGs, sustainable development goals. 2016.
3. Rudan I, Yoshida S, Chan KY, Sridhar D, Wazny K, Nair H, et al. Setting health research priorities using the CHNRI method: VII. A review of the first 50 applications of the CHNRI method. J Glob Health. 2017;7:011004.
